# QSAR Study on Antioxidant Tripeptides and the Antioxidant Activity of the Designed Tripeptides in Free Radical Systems

**DOI:** 10.3390/molecules23061407

**Published:** 2018-06-10

**Authors:** Nan Chen, Ji Chen, Bo Yao, Zhengguo Li

**Affiliations:** 1 School of Life Science, Chongqing University, Chongqing 401331, China; chennan0205@cqust.edu.cn; 2 College of Chemistry and Chemical Engineering, Chongqing University of Science and Technology, Chongqing 401331, China; jichencq@126.com (J.C.); yaobocd@foxmail.com (B.Y.); 3 Chongqing Key Laboratory of Industrial Fermentation Microorganism, Chongqing University of Science and Technology, Chongqing 401331, China

**Keywords:** antioxidant tripeptides, QSAR, multiple linear regression (MLR), support vector machine (SVM), random forest (RF), antioxidant activities

## Abstract

In this study, quantitative structure-activity relationship (QSAR) models were determined based on 91 antioxidant tripeptides. We firstly adopted the stepwise regression (SWR) method for selecting key variables without autocorrelation and then utilized multiple linear regression (MLR), support vector machine (SVM), random forest (RF), and partial least square regression (PLS) to develop predictive QSAR models based on the screened variables. The results demonstrated that all the established models have good reliability (*R*^2^*_train_* > 0.86, *Q*^2^*_train_* > 0.70) and relatively good predictability (*R*^2^*_test_* > 0.88). The contribution of amino acid residues was calculated from the stepwise regression combined with multiple linear regression (SWR-MLR) method model that shows Trp, Tyr, or Cys at C-terminus is favorable for antioxidant activity of tripeptides. Nineteen antioxidant tripeptides were designed based on SWR-MLR models, and the antioxidant activity of these tripeptides were evaluated using three antioxidant assays in free radical systems (1,1-diphenyl-2-picrylhydrazyl (DPPH) radical scavenging capacity, trolox equivalent antioxidant capacity assay, and the ferric reducing antioxidant power assay). The experimental antioxidant activities of these tripeptides were higher than the calculated/predicted activity values of the QSAR models. The QSAR models established can be used to identify and screen novel antioxidant tripeptides with high activity.

## 1. Introduction

Reactive oxygen species (ROS), such as hydroxyl radicals, superoxide anions, and hydrogen peroxide can cause oxidative damages to the cellular biomolecules and lipid peroxidation in food processing when ROS are generated in excess or are not eliminated after formation [1,2,3]. In recent years, antioxidant peptides with 2–6 amino acid residues, such as carnosine, glutathione, and so on, have been reported as novel antioxidants for applications in promoting human health and food processing [4,5]. The antioxidant peptides protect cells against oxidative stress by scavenging intracellular ROS [6], quenching free radicals [7], chelating transition metals [8,9] directly, and also improving activities of intracellular antioxidant enzymes, such as superoxide dismutase (SOD), catalase (CAT), glutathione reductase (GR), glutathione peroxidase (GP), and so on [10,11,12].

So far, hundreds of antioxidant peptides have been prepared and identified from various dietary sources [13,14,15], but the relationships between antioxidant properties and the structural characteristic of antioxidant peptides are still not fully understood. Researchers generally considered that some reducing amino acid residues in the peptides, such as His, Met, Tyr, Cys, or Trp can directly scavenge ROS [16]. Chen et al. found Pro-His-His (PHH) was the most active amino acid sequence among the 28 synthesized antioxidant peptides [17]. Saito et al. believed that tripeptides either containing Trp or Tyr residues at the C-terminus had strong radical scavenging activities [18]. Since the quantitative structure-activity relationships (QSAR) were widely used for predicting high activity peptides, like the antimicrobial, angiotensin converting enzyme (ACE) inhibitory peptides, several researchers performed the two dimensional (2D)-QSAR or three dimensional (3D)-QSAR models to understand the structural factors controlling the activity of antioxidant peptides. Li et al. used the partial least square (PLS) method to construct a QSAR model of antioxidant tripeptides with six sets of amino acid descriptors; they concluded that divided physicochemical property score (DPPS) descriptors as the amino acid descriptors are better than hydrophobic, electronic, steric, and hydrogen (HESH), vectors of hydrophobic, steric, and electronic properties (VHSE), molecular surface weighted holistic invariant molecular (MS-WHIM), isotropic surface area–electronic charge index (ISA-ECI), and *Z*-scale. They also found that N-terminal amino acid of the tripeptides should be a high hydrophobic and low electronic amino acid [19]. In another article, they collected antioxidant peptides from free radical systems and used PLS to perform the QSAR model. They found that C-terminal regions were more important than N-terminal regions for predicting antioxidant activity, and C-terminal regions with bulky hydrophobic amino acids were related to the antioxidant capability [20]. At present, many other chemometric methods like artificial neural networks, principal component analysis, random forest, and support vector machine have been employed to build QSAR models for bioactive peptides [21]. As far as we know, there are few reports of these methods applied to develop QSAR models for antioxidant peptides.

There are various antioxidant assays in vitro to evaluate the activities of peptides. These assays are approximately divided into two categories on the basis of the chemical reactions: one is single-electron-transfer (SET) reaction and the other is hydrogen-atom-transfer (HAT) reaction [5]. The 1,1-diphenyl-2-picrylhydrazyl (DPPH) radical scavenging activity, trolox equivalent antioxidant capacity (TEAC) assay, and the ferric ion reducing antioxidant power (FRAP) assay can be attributed into SET reactions and the inhibition of low-density lipoprotein autoxidation, oxygen radical absorbance capacity (ORAC), and total radical trapping antioxidant parameter (TRAP) should belong to the HAT reactions. There are also other in vitro methods to evaluate antioxidant capacity of peptides like cellular antioxidant activity assay [22,23]. However, SET reaction is the most widely used method to evaluate the antioxidant activity of peptides because of its convenience and accuracy.

The aim of this study is to develop highly predictive QSAR models that can guide the discovery of novel antioxidant tripeptides. A dataset consisting of 91 antioxidant tripeptides retrieved from the literature was adopted to study QSAR models of tripeptides. The key variables for activity of tripeptides were selected using the stepwise regression (SWR). Support vector machine (SVM), random forest (RF), multiple linear regression (MLR) and partial least square regression(PLS) were simultaneously utilized to perform QSAR models and the contribution of amino acids for antioxidant tripeptides from the models were analyzed. Then, we designed nineteen novel antioxidant tripeptides based on the established QSAR model, and we evaluated the antioxidant activity using three antioxidant assays in free radical systems (DPPH, TEAC, and FRAP).

## 2. Results and Discussions

### 2.1. QSAR Modeling and Predominant Residue Analysis

Five hundred eighty-five (195 × 3) variables were used to characterize each tripeptide. The key variables were screened by the SWR, and their autocorrelations were validated by statistical methods. With the default thresholds for SWR (*F*_1_ = 0.05 and *F*_2_ = 0.10), seven variables were screened to build QSAR models which are listed in Table 1. The variables can characterize conformations of secondary structures, electric properties, and energy of tripeptides. These screened variables were further validated by variance inflation factors (VIF), and supplementary Table S1 shows their VIF value < 2.0, which means they were irrelevant.

PLS, SVM, RF, and MLR methods were then used to develop QSAR models based on the screened variables. The statistical parameters of these methods are shown in Table 2. It shows that the four QSAR models had good reliability (Training dataset: *R*^2^*_train_* > 0.86, *Q*^2^*_train_* > 0.70) and relatively good predictability (Test dataset: *R*^2^*_test_* > 0.88) with obvious statistical significance. For the training dataset, the PLS model has the most powerful fitting capacity, and the stepwise regression combined with multiple linear regression method (SWR-MLR) model has more reliability than the others (*Q*^2^*_tain_* = 0.798). They possess similar estimating ability (*R^2^_tain_* = 0.902 vs. *R*^2^*_tain_* = 0.893). For the test dataset, the predictability of the SWR-MLR model is close to the best one built by the stepwise regression combined with random forest (SWR-RF) model *(R*^2^*_test_* = 0.897 vs. *R*^2^*_test_* = 0.914). Thus, the contributions of amino acids based on the coefficients of the SWR-MLR can be used to explore and screen novel antioxidant peptides.

The obtained linear equation for the key variable selection based on SWR-MLR was given as follows:

Y = 0.255 LEVM760107 + 0.202 COHE430101 + 0.741 CHAM820102 − 0.245 FAUJ880112 + 0.236 QIAN880115 − 0.254 YUTK870102 − 0.374 OOBM850102

Y is the antioxidant activity evaluated by the TEAC assay, *N*_train_ = 60, *R*^2^_train_ = 0.893, *R^2^_test_* = 0.897, F = 61.745, *Q^2^_LOO_* = 0.798, *Q_10-fold_* = 0.822.

**Figure 1:**
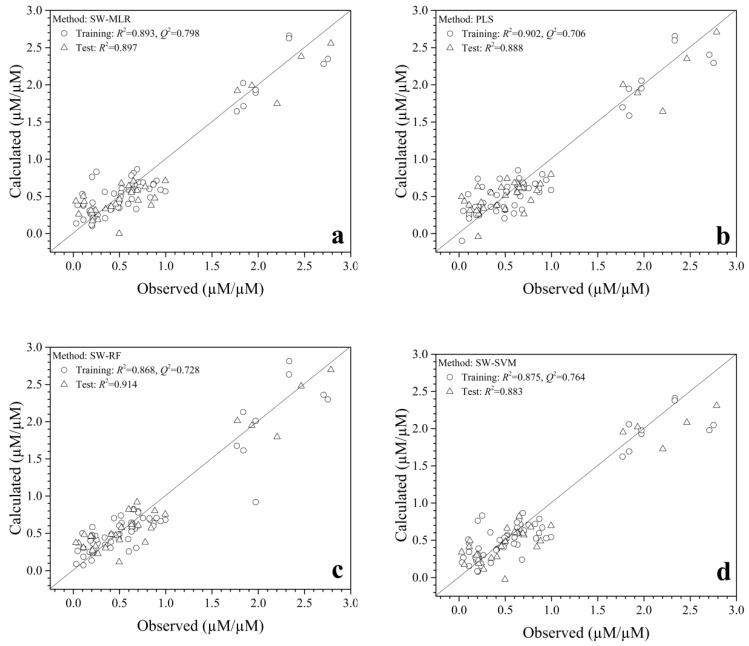
Plots of the calculated/predicted and the observed activities of antioxidant tripeptides for the (**a**) SWR-MLR model, (**b**) PLS model, (**c**) SWR-FR model, and (**d**) SWR-SVM model.

The plots of calculated/predictive and observed activities of antioxidant tripeptides are shown in Figure 1. Figure 1a,b,d show that the points obtained were scattered about the 45-degree line, which revealed the calculated/predicted activity was consistent with the observed one, which also means these QSAR models had good reliability for the training and test datasets. Interestingly, a few outlier points emerged in the training dataset for the SWR-RF model. However, Figure 1 indicates that a lot of samples have a low relative antioxidant activity (from 0.028 μM Trolox equivalent (TE)/μM to 0.995 μM TE/μM) and a small number of peptides have high activity (from 1.768 μM TE/μM to 2.2753 μM TE/μM) in the dataset, this may because of limited data available that may decrease the calculated/predictive accuracy for new samples with high antioxidant capacity. The quality would be improved if more samples with higher activity were added in the modeling.

**Figure 2:**
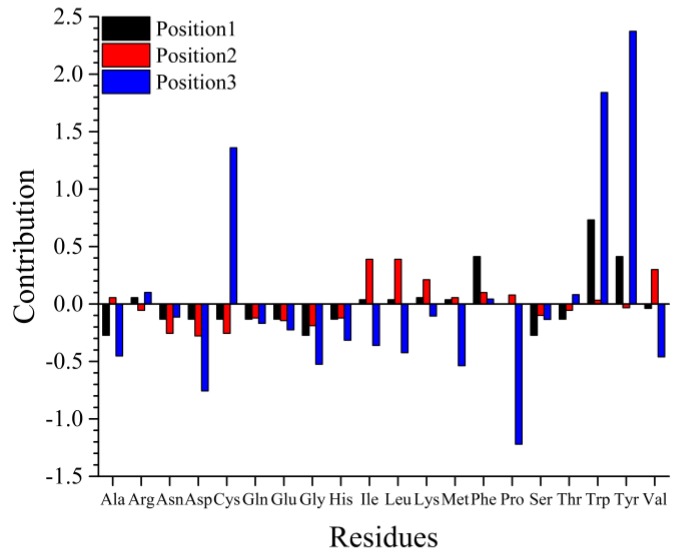
Predominant and deleterious residues at each position.

The total contribution of every amino acid at each position based on the normalized coefficients of the SWR-MLR model was calculated (Figure 2 and supplementary Table S1) to explore the favorable residue for virtual design and screening. The contributions of position 1 and 2 were associated with hydrophobic properties of residues. Figure 2 indicates Trp or Phe at position 1 were advantageous for tripeptides to form high antioxidant activity, but Ala, Gly, or Ser were unfavorable. Similarly, Ile, Met, and Val at position 2 can increase the activity of the tripeptide, but Asn, Asp, and Cys may decrease their activity. Trp, Tyr, or Cys at position 3 can denote electronic, energy, and secondary structure properties and are favorable for activity, but Pro, Asp, or Met cannot be located at this position. This means the peptides with Tyr or Trp at position 3 may have higher activity than those with Pro, Asp, or Met at position 3. For Tyr or Cys at position 3, the most important contribution came from the free energy of solution in water (CHAM820102), Trp at position 3 contributes the antioxidant activity from the optimized propensity to form reverse turn (OOBM850102) to affect the secondary structure of peptides. Based on the above results, we designed nineteen novel tripeptides and evaluated their antioxidant activity using DPPH radical scavenging capacity, trolox equivalent antioxidant capacity assay, and the ferric reducing antioxidant power assay.

### 2.2. Determination of Relative Antioxidant Activity

#### 2.2.1. DPPH Radical Scavenging Capacity

**Figure 3:**
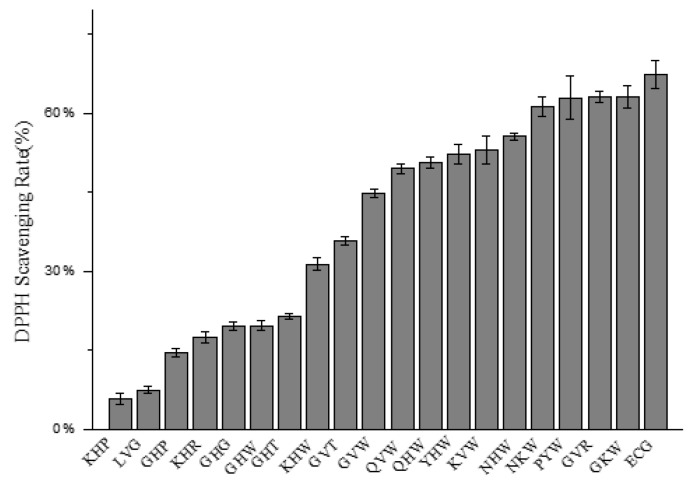
DPPH radical scavenging capacity of designed tripeptides.

DPPH radical scavenging assay was widely used in evaluating the radical scavenging activities of antioxidants because of its simplicity and rapidity [31,32,33]. The results of the DPPH radical scavenging capacity of nineteen tripeptides are shown in Figure 3. The DPPH radical scavenging rates of these tripeptides were from 5.72% to 63.13% with the concentration of 1.0 mg/mL. Figure 3 shows Gly-Lys-Trp (GKW), Gly-Val-Arg (GVR), Pro-Tyr-Trp (PYW), Asn-Lys-Trp (NKW) with the DPPH scavenging rates of 63.13%, 63.02%, 61.21%, and 55.62%, respectively. Their DPPH scavenging activities is almost equivalent to the positive control Glu-Cys-Gly (ECG). The tripeptides with Trp at the C-terminus shows high activity in the DPPH radical scavenging assay. This result is consistent with SWR-MLR models. Other researchers have found that the presence of Tyr or Trp was responsible for the antioxidant activity of peptides [34,35], especially when the Trp or Tyr residue appeared at the C-terminus [18].

#### 2.2.2. Trolox Equivalent Antioxidant Capacity (TEAC) Assay

**Figure 4:**
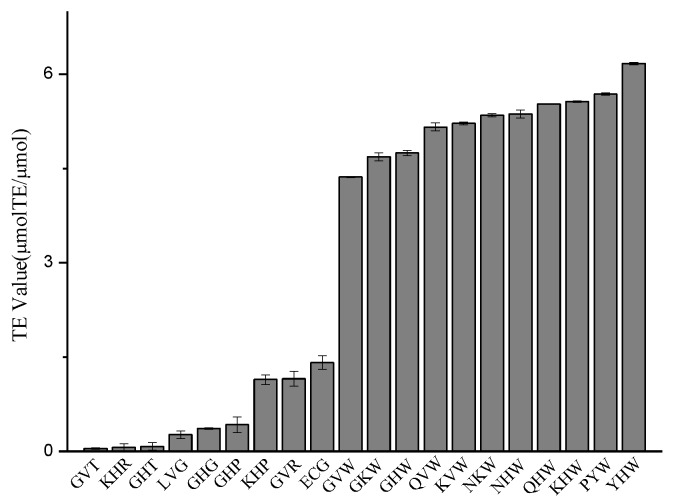
2,2′-azino-bis(3-ethylbenzthiazoline-6-sulfonic acid radical (ABTS·+) scavenging activity of tripeptides.

The TEAC results of the 19 designed tripeptides are shown in Figure 4. Tyr-His-Trp (YHW) exhibits the strongest 2,2′-azino-bis(3-ethylbenzthiazoline-6-sulfonic acid radical (ABTS +) scavenging capacity (6.169 μM TE/μM) followed by PYW (5.683 μM TE/μM), Lys-His-Trp (KHW) (5.566 μM TE/μM), Gln-His-Trp (QHW) (5.524 μM TE/μM), and Asn-His-Trp (NHW) (5.349 μM TE/μM) with the concentration of 1.0 mg/mL while the ECG exhibits the TEAC value of 1.413 μM TE/μM. These tripeptides all possesses Trp residues at the C-terminus which can act as an electron donor, while the tripeptides with low ABTS·+ radical scavenging capacity like Lys-His-Arg (KHR) and Gly-His-Thr (GHT) possess a hydrophilic amino acid at the C-terminus.

#### 2.2.3. Ferric Reducing Antioxidant Power (FRAP) Assay

**Figure 5:**
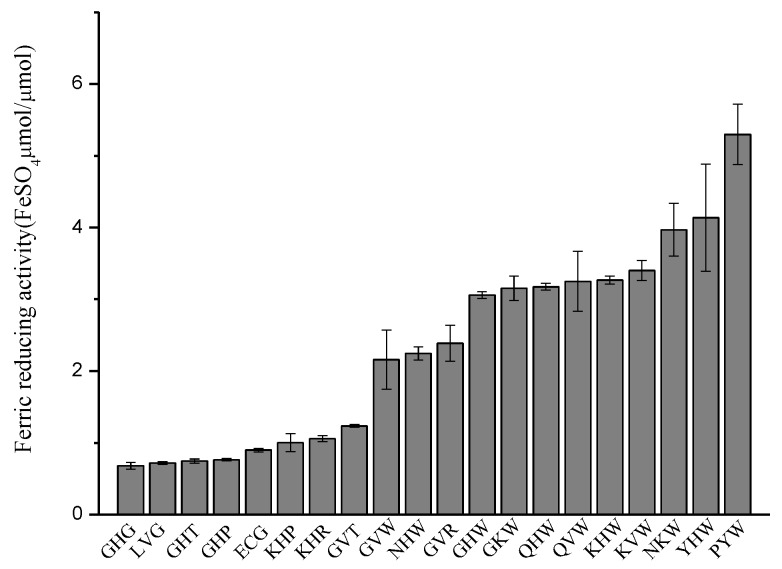
Ferric reducing activity of tripeptides.

The ferric reducing antioxidant activity of the tripeptides was measured by reducing potential of an antioxidant reaction with a 2,4,6-tripyridyl-s-triazine (TPTZ)-Fe (III) complex [36]. As Figure 5 shows, the ferric reducing capacity of the tripeptides are from 0.680 FeSO_4_ μM/μM to 5.298 FeSO_4_ μM/μM. PYW, YHW, NKW, Lys-Val-Trp (KVW), and Lys-His-Trp (KHW) display the top five high ferric reducing antioxidant abilities, and the values are 5.298 FeSO_4_ μM/μM, 4.136 FeSO_4_ μM/μM, 3.968 FeSO_4_ μM/μM, 3.401 FeSO_4_ μM/μM, and 3.270 FeSO_4_ μM/μM, respectively. The tripeptides with Trp at position 3 had higher activity than the tripeptides which have Arg, Thr, Pro, or Gly at position 3, which means the predominant residues are located at this key position. The deleterious residues at the key position will decrease their relative activity, such as Gly, Thr, and Pro at position 3. The activity of Gly-His-Gly (GHG), Leu-Val-Gly (LVG), GHT, and Gly-His-Pro (GHP) are 0.680 FeSO_4_ μM/μM, 0.719 FeSO_4_ μM/μM, 0.745 FeSO_4_ μM/μM, and 0.764 FeSO_4_ μM/μM, respectively. Moreover, if the predominant residues were superior to the deleterious ones at a key position, the relative activity would increase, just as the activity of KHW was 3.268 FeSO_4_ μM/μM. These results are in good agreement with the established SWR-MLR model.

### 2.3. Validation of QSAR Model

**Figure 6:**
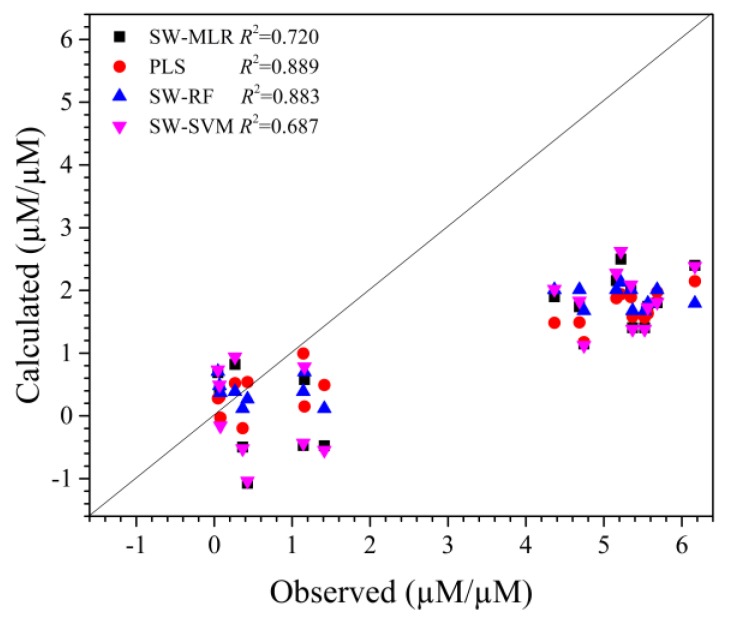
Plots for predicted and observed relative activity of designed tripeptides.

The antioxidant activity of the designed tripeptides were predicted by SWR-MLR, SWR-RF, SWR-SVM, and PLS models (Supplementary Table S2). We set the evaluated antioxidant activity of these tripeptides (TEAC assay) as observed values. The correlative coefficients between predicted values and observed values were all larger than 0.68 (Figure 6), which means that these models could be used to guide the design of antioxidant tripeptides. Compared with PLS and SWR-RF models (*R*^2^ = 0.889 vs. *R*^2^ = 0.883), SWR-MLR and SWR-SVM models have acceptable predictability for new samples. These are consistent with the results from training and test datasets, but the calculated/predicted activity of the tripeptides was underestimated compared with the observed activity, which may be due to the relatively lower activity for major samples of the dataset (0.0 μM TE/μM–1.0 μM TE/μM). These tripeptides show higher (4 μM TE/μM–6.2 μM TE/μM) antioxidant activity evaluated by TEAC, which suggests there is some unknown antioxidant activity mechanism that needs to be revealed in other antioxidant activity assays.

## 3. Materials and Methods

### 3.1. Data Source

A dataset with ninety-one synthetic antioxidant tripeptides was adopted from the literature [18,20]. The antioxidant activities of these tripeptides were tested by TEAC assay and are expressed as the TEAC value (μM TE/μM). The dataset was divided into a training database and a test dataset. Briefly, all tripeptides were sorted by their descending antioxidant activity and one in three samples was selected as the test dataset. Sixty samples in the training dataset were used to construct the QSAR model, and the leave-one-out (LOO) cross-validation method and the k-fold validation were utilized to for validation. Thirty-one samples in the test dataset were used to verify the predictability of the model. All tripeptides and their antioxidant activities are listed in supplementary Table S3.

### 3.2. Structural Characterization of Tripeptides

For a set of peptide analogues, the structures can be characterized by describing each varied amino acid with physiochemical properties. One hundred and ninety-five physiochemical properties collected from the AA Index database (http://www.genome.jp/aaindex) [37] were selected to characterize each tripeptide. Briefly, if the peptide consists of ‘*n*’ amino acids and each amino acid has ‘*m*’ properties, parameters for every peptide are ‘*n* × *m*’ elements and every peptide can be expressed as *V_jk_*, where *j* (*j* = 1…*n*) is the position of the amino acid and *k* ( *k* = 1…*m*) is the order of parameter [38,39,40].

### 3.3. Quantitative Structure-Activity Relationship Modeling

Every antioxidant tripeptide was characterized by 585 variables and the key variables for activity were screened by stepwise regression (SWR) [41]. Then, the screened variables were used to construct the QSAR model with multiple linear regressions (MLR), here named SWR-MLR. The variables screened by SWR were optimized by regulating the threshold of introducing (*F*_1_) and eliminating (*F*_2_), that can improve the *Q*^2^ of the LOO and the *R*^2^ of test dataset for MLR modeling. In order to develop an accurate QSAR model to predict the activities of the antioxidant tripeptides, the support vector machine (SVM) method and random forest (RF) method, now named SWR-SVM and SWR-RF, respectively, were used to build QSAR models based on the same variables screened by SWR. For comparison with the above modeling methods, we adopted partial least squares (PLS) to construct the QSAR model with the same variable number.

The contribution of variables for activity was calculated based on the normalized regression coefficients (NRCs), which may estimate the importance of the variables and guide the design of novel peptides. Meanwhile, the variance inflation factor (VIF) calculated by 1/(1 − *r*^2^) was used to estimate the correlation of variables, where *r*^2^ is the multiple correlation coefficient of one variable's effect regressed on the remaining variables. If the VIF value is less than 5, the variable is considered to have no correlation with other variables [42,43]. All calculations were performed using Matlab (Trial Version; Math works Inc, Natick, MA, USA) on a computer.

### 3.4. Rational Design of Antioxidant Peptides

The contribution of residues calculated from the SWR-MLR model shows Trp, Tyr, or Cys at position 3 is favorable for antioxidant capability of tripeptides. Combining with the research of Chen et al. [17], we designed nineteen tripeptides, of which eleven tripeptides had a Trp at the C-terminal and ten tripeptides had His at position 2. Considering the diversity of peptides, some unfavorable amino acids were used in the design of peptides. The designed nineteen tripeptides were used to screen antioxidant peptides with high activity and validate the reliability of constructed QSAR models (Listed in supplementary Table S2).

### 3.5. Antioxidant Activity Assay

The 1,1-diphenyl-2-picrylhydrazyl (DPPH) was purchased from Merck Life Science (Shanghai) Co., Ltd (Shanghai, China). The total antioxidant capacity assay kits for the TEAC method and the FRAP method were both purchased from Beyotime Institute of Biotechnology (Haimen, China). All the other reagents used in this research were of analytical grade and were purchased from Chengdu Chron Chemicals Co., Ltd (Chengdu, China).

Tripeptides (listed in supplementary Table S2) were synthesized by Top-peptide Co., Ltd. (Shanghai, China). The purity of the nineteen tripeptides was more than 95%.

#### 3.5.1. DPPH Radical Scavenging Activities

DPPH radical scavenging activity of each tripeptide was performed according to the method described by Musa et al. with a little modification [44]. An aliquot of 100 μL of each tripeptide solution (0.1 mg/mL) was mixed with 100 μL of DPPH solution (0.2 mM in 95% ethanol) in a 96-well microplate. The reaction mixtures were incubated for 30 min in darkness at room temperature. The absorbance of mixtures was measured at 517 nm with a microplate absorbance reader (Bio-Rad, iMark680, Hercules, CA, USA). Distilled water was used as a blank control and ECG as a positive control. The radical scavenging capacity of each tripeptide sample was measured using the following equation:

DPPH radical scavenging activity (%) = [(A_c_ − A_s_)/A_c_] × 100%

where A is absorbance, A_s_ is absorbance of sample, and A_c_ is absorbance of control.

#### 3.5.2. Trolox Equivalent Antioxidant Capacity (TEAC) Assay

The TEAC assay was performed according to the method described by Luo et al. with slight modification [36]. The ABTS·+ stock solution was prepared with 400 μL ABTS and 400 μL oxidant solution from the ABTS assay kit, then the stock solution was kept overnight in the dark at room temperature for 12~16 h to generate ABTS·+ radical cation. ABTS·+ working solution was then prepared by diluting the stock solution with phosphate buffered saline (PBS) (138 mM NaCl in 5 mM sodim phosphate buffer, pH 7.4) with an absorbance of 0.7 ± 0.05 at 734 nm. The reaction mixture contained 200 μL ABTS·+ working solution and 10 μL sample (0.1 mg/mL in PBS) or the trolox standard. PBS was a blank control. The mixture of ABTS·+ and the tripeptide was incubated at 25 °C in a 96-well microplate for 10 min, then the absorbance was measured at 734 nm by a microplate absorbance reader (Bio-Rad). The relative activity was calculated using the trolox standard solution (150μM–1500μM) calibration curve and be converted to the trolox equivalent antioxidant capacity (TEAC) value expressed as TE μM/μM tripeptide. The ECG was used as a positive control. The ABTS·+ radical scavenging activity of the sample was calculated as follows:

ABTS·+ radical scavenging activity % = [(A_c_ − A_s_)/A_c_ × 100]

where A is absorbance, A_s_ is absorbance of sample, and A_c_ is absorbance of control.

#### 3.5.3. Ferric Reducing Antioxidant Power (FRAP) Assay

The FRAP assay was performed according to the method described by Luo et al. with slight modification [36]. In the assay kit with FRAP method, stock solutions include detective buffer, 2,4,6-tripyridyl-s-triazine (TPTZ) solution, and TPTZ dilution. The working solution was prepared before use by mixing TPTZ dilution, detective buffer, and TPTZ solution in a ratio of 10:1:1 (*v*/*v*/*v*), respectively, then the working solution was incubated at 37 °C. Five µL tripeptide sample (0.1 mg/mL in PBS) was added into the TPTZ solution (180 µL) in a 96-well microplate. After incubation for 30 min at 25 °C, the absorbance of the reaction mixture was measured at 593 nm with a microplate absorbance reader (Bio-Rad). The ferric reducing activity was calculated using the FeSO_4_ solution (200 μM–1200 μM) calibration curve and was converted to the Fe^2+^ antioxidant capacity value, which was expressed as FeSO_4_ μM/μM tripeptide.

### 3.6. Statistical Analysis

All experiments were carried out in triplicate. Data were expressed as means ± standard deviation (SD). The data were analyzed by ANOVA and Duncan's new multiple range test using SPSS 18.0 (SPSS Inc., Chicago, IL, USA).

## 4. Conclusion

In this study, we have compared the performance of SVM, MLR, RF, and PLS in an antioxidant tripeptide QSAR study. The obtained results showed that all the constructed models have good reliability (*R^2^_train_* > 0.86, *Q^2^_train_* > 0.70) and predictability (*R*^2^*_test_* > 0.88); the SWR-MLR can be used to derive statistical models. The contribution of residues calculated from the SWR-MLR model shows Trp, Tyr, or Cys at position 3 is favorable for antioxidant capability of tripeptides. Nineteen novel antioxidant tripeptides were designed and predicted based on thr QSAR models. The activity of the nineteen tripeptides were furtherly evaluated by three antioxidant assays in free radical systems, and the experimental antioxidant activities of these tripeptides were higher than the calculated/predicted activity values. The established QSAR models can be used to identify and screen novel antioxidant tripeptides with high activity.

## Figures and Tables

**Table 1:** The description of selected variables used for modeling.

Name	Description
LEVM760107	van der Waals parameter [24]
COHE430101	Partial specific volume [25]
CHAM820102	Free energy of solution in water, kcal/mole [26]
FAUJ880112	Negative charge [27]
QIAN880115	Weights for beta-sheet at the window position of -5 [28]
YUTK870102	Unfolding Gibbs energy in water, pH9.0 [29]
OOBM850102	Optimized propensity to form reverse turn [30]

**Table 2:** The statistical parameters of the quantitative structure-activity relationship (QSAR) models.

Methods	Training Dataset											Test Dataset	
	*n*	*m*	*R* ^2^ *_train_*	SD	*F*		*Q* ^2^_LOO_	*Q* ^2^_4-fold_	*Q* ^2^_6-fold_	*Q* ^2^_10-fold_	PRESS	*n*	*R* ^2^ *_test_*
PLS	60	7	0.902	0.226		68.005	0.706	0.749	0.700	0.708	7.891	31	0.888
SWR-SVM	60	7	0.875	0.254		45.820	0.764	0.764			6.327	31	0.883
SWR-RF	60	7	0.868	0.261		48.857	0.728	0.728			7.298	31	0.914
SWR-MLR	60	7	0.893	0.235		61.745	0.798	0.792	0.802	0.822	5.413	31	0.897

Note: *n* is referring the number of the training dataset or the test dataset, *m* is the number of the key variable, *R^2^* is the squared correlation coefficient, SD is the standard deviation, *F* is referring the *F* statistical value, and *Q^2^* is correlation coefficient from leave-one-out (LOO) or k-fold validation, PRESS is the predicted residual error sum of squares, PLS is partial least square regression, SWR-SVM is the stepwise regression combined with support vector machine, SWR-RF is the stepwise regression combined with random forest, and SWR-MLR is the stepwise regression combined with multiple linear regression.

## Supplementary Materials

Supplementary material is available on line, Table S1: The contribution of each amino acid (AA) for activity, Table S2: The observed (Obs.) and calculated/predicted relative antioxidant activity for designed tripeptides. Table S3: The observed (Obs.) and calculated/predicted activity from different models.
