# Supplementary material for: QSAR Study on Antioxidant Tripeptides and the Antioxidant Activity of the Designed Tripeptides in Free Radical Systems
Source: Molecules. 2018 Jun 10;23(6):1407. doi: 10.3390/molecules23061407 (PMC6100293; doi:10.3390/molecules23061407)
Supplement: Supplementary file 1 [file molecules-23-01407-s001.pdf]

Supporting information

QSAR Study on Antioxidant Tripeptides and the Antioxidant Activity of the Designed Tripeptides in Free Radical Systems

Nan Chen [1,2,3], Ji Chen [2,3] , Bo Yao [2,3] and Zhengguo Li [1,*]

[1] School of life science, Chong Qing University, Chong Qing 401331, China; chennan0205@cqust.edu.cn

[2] College of Chemistry and Chemical Engineering, Chong Qing University of Science and Technology, Chong Qing 401331, China; jichencq@126.com (J.C.); yaobocd@foxmail.com (B.Y.)

[3] Chongqing Key Laboratory of Industrial Fermentation Microorganism, Chong Qing University of Science and Technology, Chong Qing 401331, China

* Correspondence: zhengguoli@cqu.edu.cn

Table S1. The contribution of each amino acid (AA) for activity. VIF = variance inflation factor.

AA	LEVM-760107	COHE-430101	CHAM-820102	FAUJ-880112	QIAN-880115	YUTK-870102	OOBM-850102
VIF	1.105	1.105	1.436	1.681	1.335	1.379	1.706
Pos	1	2	3	3	3	3	3
Ala	-0.273	0.056	-0.488	0.080	-0.025	0.061	-0.080
Arg	0.056	-0.056	-0.778	0.080	0.003	0.782	0.014
Asn	-0.132	-0.256	-0.326	0.080	0.364	0.125	-0.357
Asp	-0.132	-0.278	0.577	-0.717	-0.099	0.040	-0.557
Cys	-0.132	-0.256	1.659	0.080	-0.266	-0.098	-0.015
Gln	-0.132	-0.122	-0.006	0.080	-0.007	-0.119	-0.116
Glu	-0.132	-0.145	0.449	-0.717	0.068	0.263	-0.287
Gly	-0.273	-0.189	-0.557	0.080	0.105	0.103	-0.256
His	-0.132	-0.122	-0.326	0.080	0.188	-0.194	-0.063
Ile	0.037	0.389	0.020	0.080	-0.266	-0.278	0.084
Leu	0.037	0.389	0.143	0.080	-0.248	-0.512	0.113
Lys	0.056	0.211	-0.326	0.080	0.058	-0.013	0.096
Met	0.037	0.056	-0.039	0.080	-0.544	-0.109	0.074
Phe	0.413	0.100	0.138	0.080	-0.127	-0.098	0.050
Pro	-0.001	0.078	-1.308	0.080	0.225	0.050	-0.268
Ser	-0.273	-0.100	-0.556	0.080	0.392	-0.066	0.016

Thr	-0.132	-0.056	-0.326	0.080	0.309	0.040	-0.020
Trp	0.732	0.033	0.375	0.080	-0.155	0.178	1.363
Tyr	0.413	-0.033	1.826	0.080	0.123	0.061	0.284
Val	-0.038	0.300	-0.151	0.080	-0.099	-0.215	-0.075

Table S2. The observed (Obs.) and calculated/predicted relative antioxidant activity for designed tripeptides.

Seq.		Obs.	SWR-MLR	PLS	SWR-RF	SWR-SVM
Glu-Cys-Gly	(ECG)	1.413	-0.478	0.492	0.116	-0.546
Gly-His-Gly	(GHG)	0.365	-0.498	-0.195	0.116	-0.523
Gly-His-Pro	(GHP)	0.426	-1.074	0.539	0.269	-1.039
Gly-His-Thr	(GHT)	0.079	-0.063	-0.028	0.371	-0.159
Gly-His-Trp	(GHW)	4.745	1.146	1.176	1.676	1.124
Gly-Lys-Trp	(GKW)	4.687	1.740	1.490	2.012	1.830
Gly-Val-Arg	(GVR)	1.157	0.579	0.151	0.700	0.782
Gly-Val-Thr	(GVT)	0.047	0.689	0.279	0.707	0.734
Gly-Val-Trp	(GVW)	4.365	1.898	1.483	2.012	2.018
Lys-His-Pro	(KHP)	1.143	-0.473	0.996	0.387	-0.434
Lys-His-Arg	(KHR)	0.067	0.428	0.302	0.482	0.493
Lys-His-Trp	(KHW)	5.566	1.747	1.633	1.794	1.729
Lys-Val-Trp	(KVW)	5.218	2.499	1.940	2.130	2.622
Leu-Val-Gly	(LVG)	0.266	0.821	0.522	0.389	0.940
Asn-His-Trp	(NHW)	5.368	1.404	1.581	1.676	1.383
Asn-Lys-Trp	(NKW)	5.349	1.998	1.895	2.012	2.089
Pro-Tyr-Trp	(PYW)	5.683	1.803	1.975	2.012	1.813
Gln-His-Trp	(QHW)	5.524	1.404	1.568	1.676	1.383
Gln-Val-Trp	(QVW)	5.161	2.156	1.875	2.012	2.277
Tyr-His-Trp	(YHW)	6.169	2.400	2.148	1.794	2.385

Table S3. The observed (Obs.) and calculated/predicted activity from different models.

Seq.		Obs.	SWR-MLR	PLS	SWR-RF	SWR-SVM
			Training dataset			
Leu-His-Tyr	(LHY)	2.753	2.349	2.294	2.299	2.049
Pro-His-Tyr	(PHY)	2.707	2.280	2.405	2.362	1.980
Arg-Trp-Tyr	(RWY)	2.334	2.660	2.655	2.815	2.412
Leu-Trp-Tyr	(LWY)	2.332	2.626	2.598	2.635	2.378
Trp-Pro-Leu	(WPL)	1.972	1.895	2.055	0.919	1.930
Val-Pro-Trp	(VPW)	1.972	1.932	1.953	2.012	1.979
Leu-His-Trp	(LHW)	1.840	1.713	1.586	1.613	1.694
Arg-Trp-Trp	(RWW)	1.837	2.024	1.948	2.130	2.058

Pro-His-Trp	(PHW)	1.768	1.644	1.698	1.676	1.625
Arg-Trp-His	(RWH)	0.995	0.568	0.586	0.681	0.544
Pro-Trp-Asn	(PWN)	0.943	0.590	0.722	0.663	0.529
Pro-Trp-Thr	(PWT)	0.900	0.712	0.798	0.707	0.671
Leu-Trp-Arg	(LWR)	0.869	0.670	0.559	0.637	0.788
Leu-Trp-Asn	(LWN)	0.866	0.659	0.610	0.600	0.598
Pro-Trp-Ile	(PWI)	0.832	0.485	0.664	0.681	0.526
Pro-Trp-Arg	(PWR)	0.822	0.602	0.670	0.700	0.719
Arg-Trp-Lys	(RWK)	0.753	0.687	0.610	0.704	0.707
Arg-Trp-Ile	(RWI)	0.702	0.588	0.610	0.798	0.629
Arg-Trp-Asn	(RWN)	0.702	0.693	0.667	0.781	0.632
Arg-Trp-Phe	(RWF)	0.689	0.865	0.684	0.594	0.866
Lys-His-Ser	(LHS)	0.680	0.330	0.323	0.304	0.243
Arg-Trp-Glu	(RWE)	0.663	0.664	0.504	0.560	0.710
Arg-Trp-Thr	(RWT)	0.651	0.815	0.744	0.824	0.775
Pro-Trp-Gln	(PWQ)	0.637	0.608	0.851	0.638	0.592
Pro-Trp-His	(PWH)	0.632	0.465	0.641	0.563	0.440
Leu-Trp-Lys	(LWK)	0.629	0.653	0.552	0.523	0.672
Leu-Trp-Thr	(LWT)	0.627	0.781	0.687	0.644	0.740
Arg-His-Phe	(RHF)	0.600	0.588	0.380	0.258	0.537
Leu-Trp-Ala	(LWA)	0.594	0.399	0.272	0.422	0.455
Leu-Trp-Ser	(LWS)	0.522	0.607	0.627	0.640	0.572
Leu-Trp-Leu	(LWL)	0.515	0.545	0.557	0.738	0.558
Arg-Trp-Val	(RWV)	0.510	0.499	0.566	0.589	0.519
Pro-Trp-Met	(PWM)	0.498	0.345	0.318	0.490	0.465
Arg-Trp-Ala	(RWA)	0.497	0.434	0.329	0.602	0.490
Leu-Trp-Met	(LWM)	0.490	0.414	0.206	0.427	0.534
Arg-Trp-Asp	(RWD)	0.485	0.354	0.357	0.484	0.406
Pro-Trp-Ser	(PWS)	0.440	0.539	0.738	0.703	0.503
Pro-Trp-Ala	(PWA)	0.414	0.331	0.383	0.485	0.386
Leu-Trp-Gly	(LWG)	0.406	0.346	0.519	0.389	0.376
Leu-Trp-Asp	(LWD)	0.402	0.320	0.300	0.303	0.372
Pro-His-Ile	(PHI)	0.344	0.208	0.360	0.345	0.197
Pro-Trp-Gly	(PWE)	0.339	0.561	0.558	0.443	0.606
Pro-Trp-Asp	(PWD)	0.262	0.251	0.411	0.366	0.302
Leu-Trp-Phe	(LWF)	0.250	0.831	0.627	0.413	0.832
Pro-His-Leu	(PHL)	0.238	0.199	0.364	0.465	0.160
Leu-His-Ile	(LHI)	0.217	0.276	0.248	0.282	0.266
Pro-His-Arg	(PHR)	0.211	0.325	0.366	0.364	0.390
Arg-His-Asn	(RHN)	0.208	0.416	0.363	0.445	0.303
Arg-His-Leu	(RHL)	0.206	0.302	0.310	0.583	0.263
Arg-His-Gly	(RHG)	0.203	0.103	0.272	0.233	0.081
Pro-Trp-Phe	(PWF)	0.202	0.762	0.738	0.476	0.763
Pro-His-Val	(PHV)	0.198	0.119	0.316	0.136	0.087

Leu-His-Leu	(LHL)	0.186	0.268	0.253	0.402	0.229
Pro-His-Lys	(PHK)	0.176	0.307	0.360	0.250	0.274
Leu-His-Lys	(LHK)	0.108	0.376	0.248	0.187	0.343
Leu-His-Arg	(LHR)	0.108	0.393	0.255	0.301	0.459
Leu-His-Val	(LHV)	0.108	0.187	0.205	0.073	0.156
Leu-Trp-His	(LWH)	0.098	0.533	0.529	0.500	0.509
Leu-His-Asn	(LHN)	0.046	0.382	0.306	0.264	0.269
Leu-His-Met	(LHM)	0.031	0.137	-0.098	0.091	0.205
			Test Dataset			
Pro-Trp-Tyr	(PWY)	2.785	2.557	2.709	2.698	2.309
Arg-His-Tyr	(RHY)	2.464	2.383	2.351	2.480	2.083
Arg-His-Trp	(RHW)	2.203	1.747	1.643	1.794	1.729
Leu-Trp-Trp	(LWW)	1.931	1.990	1.890	1.949	2.023
Pro-Trp-Trp	(PWW)	1.774	1.921	2.002	2.012	1.954
Arg-Trp-Gln	(RWQ)	0.995	0.711	0.797	0.756	0.696
Pro-Trp-Leu	(PWL)	0.880	0.476	0.668	0.801	0.489
Arg-Trp-Gly	(RWG)	0.842	0.380	0.576	0.569	0.410
Leu-Trp-Glu	(LWE)	0.777	0.629	0.446	0.380	0.675
Arg-Trp-Met	(RWM)	0.702	0.448	0.263	0.608	0.568
Arg-Trp-Leu	(RWL)	0.689	0.579	0.614	0.919	0.593
Arg-Trp-Arg	(RWR)	0.651	0.705	0.616	0.818	0.822
Pro-Trp-Lys	(PWK)	0.634	0.584	0.664	0.586	0.603
Lew-Trp-Ile	(LWI)	0.628	0.554	0.553	0.618	0.595
Arg-Trp-Ser	(RWS)	0.600	0.642	0.684	0.821	0.606
Leu-Trp-Gln	(LWQ)	0.519	0.677	0.740	0.575	0.661
Leu-Trp-Val	(LWV)	0.499	0.464	0.509	0.409	0.485
Pro-His-Glu	(PHG)	0.496	0.000	0.327	0.116	-0.023
Pro-Trp-Val	(PWV)	0.457	0.396	0.621	0.472	0.416
Arg-His-Ser	(RHS)	0.409	0.365	0.380	0.485	0.277
Pro-His-Gln	(PHQ)	0.348	0.331	0.547	0.302	0.263
Pro-His-His	(PHH)	0.266	0.187	0.336	0.228	0.111
Pro-His-Asn	(PHN)	0.240	0.313	0.418	0.327	0.200
Arg-His-Val	(RHV)	0.212	0.222	0.262	0.254	0.190
Arg-His-Met	(RHM)	0.207	0.171	-0.041	0.272	0.239
Pro-Trp-Gly	(PWG)	0.203	0.277	0.631	0.452	0.307
Arg-His-Ile	(RHI)	0.189	0.311	0.306	0.462	0.300
Arg-His-Arg	(RHR)	0.118	0.428	0.312	0.482	0.493
Leu-His-Thr	(LHT)	0.108	0.504	0.383	0.308	0.411
Pro-His-Ser	(PHS)	0.058	0.262	0.434	0.367	0.174
Pro-His-Thr	(PHT)	0.028	0.435	0.494	0.371	0.342
